# The Central Cholinergic Synapse: A Primer

**DOI:** 10.3390/ijms26199670

**Published:** 2025-10-03

**Authors:** Jochen Klein

**Affiliations:** Department of Pharmacology and Clinical Pharmacy, FB14, J. W. Goethe University of Frankfurt, Max-von-Laue-Str. 9, D-60438 Frankfurt, Germany; klein@em.uni-frankfurt.de; Tel.: +49-6131-540-3803

**Keywords:** acetylcholine, glucose, choline, choline acetyltransferase, vesicular acetylcholine transporter, high-affinity choline uptake, acetylcholinesterase

## Abstract

The central cholinergic system is an important player in the control of motor function, appetite, the reward system, attention, memory and learning. Its participation in neurological diseases (e.g., Alzheimer’s and Parkinson’s disease, epilepsy) and in psychiatric diseases (e.g., schizophrenia, depression) makes it a preferred study subject for drug development. The present review summarizes salient features of the central cholinergic synapses that will guide future studies. Cholinergic synapses are defined by the presence of choline acetyltransferase (ChAT), the vesicular ACh transporter (VAChT), the high-affinity choline transporter CHT-1 and the presence of PRiMA-coupled acetylcholinesterase (AChE). The firing frequency of cholinergic fibers is reflected in high-affinity choline uptake activity, which also responds to variations in ChAT, VAChT and AChE activities conferring considerable plasticity to cholinergic responses. The availability of glucose and choline can limit ACh synthesis and release under conditions of high ACh turnover. Future studies will focus on rapid methods to measure ACh release and a deeper understanding of cholinergic plasticity during development, aging and dementia.

## 1. Introduction

Central cholinergic pathways are often in the focus of neuroscience because they are associated with a wide variety of brain functions and diseases [1,2,3]. Central cholinergic nuclei include two groups that contain projection neurons to other parts of the brain. The forebrain cholinergic nuclei (classified as Ch1-Ch4 nuclei) are located in the medial septum, diagonal band of Broca and the Nucleus basalis of Meynert and project to hippocampus and all cortical areas. These neurons are large and heavily branched. They dependent on nerve growth factor (NGF) and degenerate early in Alzheimer’s disease [4]. Cholinergic nuclei in the brain stem are classified as Ch5-Ch8 and project into the thalamus and other forebrain structures including the forebrain cholinergic nuclei. Cholinergic interneurons are prominent in the striatum (caudate nucleus and putamen) [4]. Cholinergic interneurons in the striatum contribute to motor control whereas cholinergic projection neurons in the basal forebrain take part in cognitive and attentional functions [2]. Cholinergic fibers originating in the pontine formation of the brain stem control REM sleep and are part of the reticular activating system that coordinates the sleep–wake rhythm. Many other functions have been ascribed to central cholinergic pathways, such as control of mood, participation in epileptic seizures and in drug addiction; in fact, hardly any brain function is known that is not influenced, at least in part, by cholinergic activity [3]. In the light of these functions, an understanding of the cholinergic synapse is of paramount importance for biomedical research. While the field moves forward with new techniques and approaches, such as novel biosensors or bioinformatics, the present paper summarizes key findings on the neurochemistry of cholinergic synapses to help orient newcomers to the field.

## 2. The Cholinergic Phenotype

Central cholinergic neurons express a limited number of specific cholinergic proteins, including choline acetyltransferase (ChAT), the enzyme that synthesizes acetylcholine (ACh); the choline carrier CHT-1, which mediates high-affinity choline uptake (HACU); and the vesicular ACh transporter VAChT [1,2,3]. In the brain, cholinergic neurons also express the majority of acetylcholinesterase (AChE), the enzyme that terminates cholinergic action, whereas butyrylcholinesterase (BChE), an enzyme that can act as a backup for AChE, is expressed by glial cells. The gene for VAChT is included in the first exon of ChAT, and both proteins show a similar developmental pattern, hence this arrangement is called the “cholinergic gene locus” [5]. The ontogeny of cholinergic neurons and the transcription factors involved in the expression of the cholinergic phenotype have been studied in some detail but are not discussed here (for review, see [6,7]).

While AChE is widely distributed in the brain and has been used to map neuronal pathways [8], mainly ChAT has been used to localize ACh-synthesizing cells in the body; selective antibodies to ChAT and GFP-coupled ChAT have been used in these endeavors [9]. It should be noted that prominent ChAT-positive cells in the periphery include cholinergic neurons in the autonomic nervous system and spinal neurons that control movement. Moreover, several types of non-neuronal cells that form and secrete ACh have been described [10]; however, in the brain, the presence of ChAT identifies a cholinergic neuron.

### 2.1. Synthesis and Turnover of ACh

**Figure 1:**
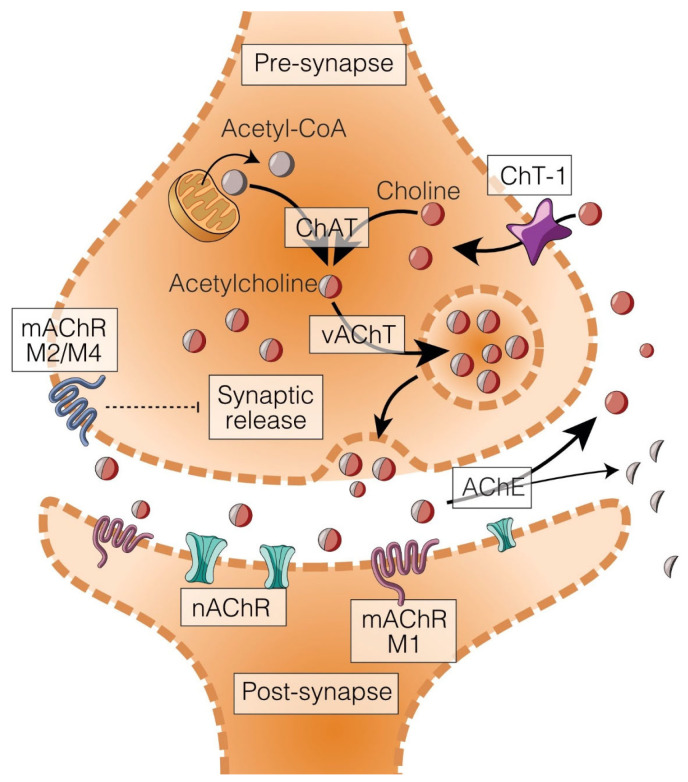
The cholinergic synapse. Choline acetyltransferase (ChAT) synthesizes acetylcholine (ACh) from acetyl-CoA (delivered from mitochondria via glycolysis) and choline (taken up from extracellular space). Low-affinity choline carriers are ubiquitous while a high-affinity carrier (HACU) mediates choline uptake specifically for ACh synthesis. ACh is transported into synaptic vesicles by a proton-dependent transporter (the vesicular ACh transporter, VAChT), and vesicles are released upon depolarization and calcium influx. Freshly released ACh interacts with muscarinic (mAChR) and nicotinic (nAChR) receptors pre- and postsynaptically. Hydrolysis of ACh by acetylcholinesterase (AChE) terminates the action of ACh.

ChAT forms acetylcholine (ACh), the cholinergic transmitter, from acetyl-CoA and choline (Figure 1). ChAT isoforms reside in the cytoplasm but can also be nuclear- or membrane-associated. Their expression follows cholinergic pathways; in the brain, ChAT expression levels are high in the striatum, moderate in the hippocampus and cortex and low in the cerebellum. ChAT exists in various splice variants, and several phosphorylation events that have modest effects on overall activity [11,12]. ChAT has relatively high Km values for its substrates, acetyl-CoA (ca. 10 μM) and choline (ca. 400 μM). These values are higher than the intracellular levels of acetyl-CoA (around 5 μM) and choline (estimated at 50 μM); therefore, an increase in substrate availability in the synaptic ending leads to an increase in ACh synthesis. In other words, ChAT activity is not considered rate-limiting for ACh synthesis [13]. In experimental studies, strong elevations of ChAT activities are observed during brain development but are unlikely in adults, whereas reductions in ChAT expression or activity in the brain are usually indicative of cholinergic cell death, e.g., in stroke or dementia.

The availability of precursors can be rate-limiting for ACh synthesis, especially because ACh has a very high turnover rate (see below). Unlike most transmitters, ACh is inactivated by enzymatic cleavage (AChE) in the synaptic cleft. Therefore, it must be continuously re-synthesized in cholinergic terminals. The turnover rate of ACh has been estimated at 6 nmol/g brain tissue per minute [14]. As the brain contains only 20 nmol/g ACh, the total pool of ACh is replaced within little more than three minutes. This turnover rate for ACh is at least 30-fold higher than that for catecholamines, which are inactivated by cellular uptake and recycled [14]. Hence, the availability of ACh precursors is pivotal for cholinergic function.

The complete knockout of the ChAT gene is lethal in mice, mainly because skeletal muscle function fails, e.g., for breathing. However, hemizygous mice develop quite normally and only show limited performance in motor exercises. In the brain of hemizygous mice, ACh synthesis is normal, but increased HACU activities point to a compensatory increase in cholinergic firing to sustain normal ACh levels [15]. In humans, mutations in ChAT, but also in the genes for AChE and the nicotinic receptor of skeletal muscle, are associated with congenital myasthenic syndromes [16].

### 2.2. Precursors: Acetyl-CoA

Acetyl-CoA in the brain is mainly formed by glycolysis. Glucose is the major fuel of the brain [17]. The brain has only limited stores of glycogen in astrocytes and does not sustain gluconeogenesis, hence glucose must be taken up from blood plasma [18]. Its plasma concentration is 4–6 mM, and it is transported into the brain by glucose transporter 1 (GLUT1), which is located at brain endothelial cells and has a very high Km value (11 mM; [19]); i.e., increases in plasma glucose cause increased influx of glucose into the brain. However, brain extracellular levels of glucose are only 1–2 mM due to rapid cellular uptake of glucose [20]. While glucose can enter neurons directly through the GLUT3 transporter, much glucose is taken up by astrocytes via the GLUT1 transporter and is converted to lactate through glycolysis. Lactate freely leaves the astrocyte through MCT transporters and can be taken up by neurons and used for energy production [21]. While glycolysis occurs in the cytoplasm, acetyl-CoA is formed from pyruvate in mitochondria and must be transported back into the cytosol. Acetyl-CoA reaches the cytosol with the help of certain carriers, i.e., acetyl-CoA is fused with oxaloacetate to citrate in the mitochondria, exported as citrate, and citrate is cleaved in the cytoplasm by ATP-citrate lyase to yield acetyl-CoA [13]. In addition, acetate (which is formed by AChE) can be taken up by cholinergic neurons, but the relevance of this pathway is probably low in mammalian brain [13].

Pyruvate oxidation to acetyl-CoA is an efficient process in mitochondria, hence glucose availability and acetyl-CoA formation are rarely rate-limiting for ACh synthesis. During cerebral ischemia, ACh synthesis is reduced; however, this may be due to general energy failure [18]. Severe, isolated hypoglycemia induced by insulin injections did not affect ACh release. Glucose can become limiting for cholinergic function when ACh synthesis is increased, e.g., during behavioral stimulation of cholinergic fibers [22,23]. When rats performed a demanding cognitive task, glucose levels in the hippocampus were reduced and ACh release was increased by glucose administration [24]. These studies show that the availability of glucose can vary and may influence ACh synthesis under stimulatory conditions.

### 2.3. Precursors: Choline

ACh and choline are minor choline metabolites with respect to abundance in the brain. The brain contains approx. 25,000 nmol/g choline bound in phospholipids (mainly phosphatidylcholine, PC and sphingomyelin, SM) and about 1000 nmol/g in soluble cytoplasmic choline metabolites, mainly phosphocholine (PCh) and glycerophosphocholine (GPC) [25]. In comparison, the brain concentrations of choline are ca. 30 nmol/g and of ACh approx. 20 nmol/g [25]. These numbers already suggest that choline and ACh levels are dynamically regulated, with phospholipids as back-up to supply additional choline when needed [26].

One note of caution is required. Functional magnetic resonance imaging (fMRI) of the brain shows a “choline” peak that is often misinterpreted in the literature. In fact, neither the immobile phospholipids nor the minute amounts of choline and ACh contribute much to this signal. Instead, the cytosolic metabolites phosphocholine (ca. 300 µM) and glycerophosphocholine (GPC; ca. 600 µM) represent the majority of the “choline” signal in MRI imaging [25,27]. Phosphocholine is formed both during phospholipid synthesis and breakdown. GPC, in contrast, is only formed during the breakdown of PC which is initiated by phospholipase A_2_ activation. GPC levels can be used as a marker of PC breakdown [27], and GPC levels are increased in neurodegenerative disease due to extensive membrane breakdown [28].

Choline has a plasma concentration of approx. 10 µM; depending on dietary intake, this value can range from 5 µM (choline-deficient diet) to 40 µM after a choline-rich meal. Choline is not considered a vitamin because it can be synthesized in the liver (by threefold methylation of phosphatidylethanolamine, PE, yielding phosphatidylcholine, PC), and choline can be released from PC by the action of phospholipases. However, choline synthesis depends on a sufficient supply of C1-bodies and folate, and a choline-deficient diet produces fatty liver disease and, ultimately, liver cancer [26,29]. The brain does not synthesize choline in appreciable amounts and is dependent on choline uptake from blood. It should be noted that much recent work on choline has focused on its role in brain development, and supplementation of choline during pregnancy and lactation was reported to enhance brain function in the adult [30].

The entry of choline into the brain occurs via a transporter at the blood–brain barrier which has a high Kd; i.e., it transports choline more rapidly when plasma choline levels rise [19]. The brain takes up choline when choline levels are high (>14 µM in the rat) but releases choline between meals when plasma choline levels are low [20]. Free choline levels in the brain extracellular space are relatively low at 3–5 µM, and they do not change much even when large doses of choline are administered. The main reason for this phenomenon is the rapid cellular uptake and subsequent phosphorylation of choline [20,26]. Due to its quaternary nitrogen atom, choline has a permanent positive charge. As a cation, it is drawn into cells which carry a negative membrane potential. In nerve cells, at −70 mV, choline would be expected to reach a 16-fold higher intracellular vs. extracellular concentration according to the Nernst equation [13]. Interestingly, the concentration of free choline (approx. 20 nmol/g brain) suggests a cytosolic concentration of 50–60 µM which is indeed 12–15-fold higher than the extracellular concentration of approx. 4 µM [31]. The dependence of choline dynamics on membrane potential is illustrated by the fact that choline is rapidly released from synaptosomes when the membrane potential is reduced by, for instance, KCl [32].

Choline enters all brain cells through transport systems—low-affinity choline uptake (LACU)—without requiring an additional energy source. Choline that is taken up by the LACU is mainly used for the synthesis of choline-containing phospholipids such as PC (brain concentration: 20 µmol/g) and SM (4–5 µmol/g). Cholinergic neurons additionally express a high-affinity uptake system (HACU; see below). As the concentration of phospholipids and other choline metabolites in CSF is low, free choline is the only compound that moves freely through the barriers such as endothelia and ependymal cells [25].

The metabolic constraints ensure that free choline is dynamically regulated between choline released from ACh and choline released from phospholipids. The latter can occur through the activation of phospholipases D1 and 2 which respond to a variety of receptors and other stimuli [33]. Under pathological conditions (e.g., convulsions, ischemia), phospholipase A_2_ is activated and breaks down PC and other phospholipids, forming GPC and free choline [27]. As ACh turnover is much more rapid than phospholipid turnover, the release of choline from ACh (approx. 6 nmol/g/min) is of the same magnitude as that from PC whereas SM in myelin is turned over rather slowly with a half-life of more than two weeks.

### 2.4. Choline and ACh Release

Acute administration of choline increases ACh release in the striatum but does not affect hippocampal or cortical levels. However, choline administration also increases hippocampal and cortical ACh release under conditions of rapid turnover [34]. When ACh release was stimulated by pharmacological or behavioral means (e.g., by atropine, scopolamine or seizures), precursor loading through a choline-rich diet as well as acute choline supplementation increased ACh release in microdialysis studies (see [34] and references cited therein). On the other hand, a choline-deficient diet reduced hippocampal, but not striatal ACh release after scopolamine injection [35]. Moreover, when plasma choline is drastically reduced by the intravenous injection of choline oxidase, clear-cut reductions in ACh release can be seen [36]. To summarize, choline levels are kept rather constant in the brain through various metabolic and transport mechanisms, but effects of choline availability on ACh release can be demonstrated during stimulated release of ACh.

The local dynamics of free choline in the synaptic area are not fully understood. In striatum, an area of relatively dense cholinergic innervation, the levels of choline fall when ACh is released and vice versa [37]. However, in hippocampus and cortex, this balance is not observed, and, in fact, choline levels often fall much more strongly than would be expected from the increase in ACh [31]. Here, choline levels may be (slowly) supplemented by phospholipid hydrolysis, and, in fact, phospholipase D activity (which liberates free choline from PC) was shown to be stimulated by various receptors including muscarinic ACh receptors [33]; thus, ACh may mobilize its own precursor, albeit in a slow fashion. In the vicinity of the cholinergic synapse, the recycling of choline from ACh predominates as is shown by the fact that hemicholinium-3 (HC-3), an effective inhibitor of HACU, almost completely blocks ACh release [38]. The high efficiency of choline uptake in the perisynaptic area was demonstrated using choline biosensors. Newly released choline is cleared from the extracellular space within seconds (at approx. 2.3 µM/s), and this clearance is significantly delayed when HACU is blocked [39].

### 2.5. High-Affinity Choline Uptake (HACU)

As a permanently charged molecule, choline can enter cells exclusively through membrane-bound choline transporters. High- and low-affinity carriers for choline have been described [40]. The low-affinity uptake system is present in all cells mainly to supply choline for phospholipid synthesis; the responsible transporter(s) have a Kd for choline of approx. 30 µM. They belong to the SLC family (e.g., SLC44A1), particularly the organic cation (OCT) transporter family [41]. The recently described transporter FLVCR may be the elusive low-affinity choline carrier at the blood–brain barrier [26].

The HACU system, represented by the choline transporter CHT-1 (SLC5A7) [42], is strictly sodium-dependent which means that it uses ATP-dependent processes—in addition to choline’s charge—to transport choline into the synaptic cytosol against a concentration gradient [43]. The transport mechanism has recently been described in molecular detail [44]. The CHT-1 transporter has a very high affinity for choline (Kd = 1–2 µM) and guarantees that choline—and especially the choline released locally by the action of acetylcholinesterase (AChE)—is preferentially imported into cholinergic nerve endings for the ongoing synthesis of ACh.

The activity of the high-affinity choline transporter, just like ChAT, is modulated by phosphorylation but, more importantly, CHT-1 shows a peculiar behavior because it shuttles between transmitter vesicles (inactive state) and cell membrane (active state) [45]. Cholinergic synaptic vesicles carry CHT-1 in their membrane, and when they fuse with the plasma membrane, some CHT-1 molecules remain there and subsequently transport choline into the synapse. When the firing rate is high, more CHT-1 accumulates in the plasma membrane, increasing choline import, whereas if the firing rate is lowered, CHT-1 molecules slowly return to intracellular vesicles by clathrin-dependent endocytosis where they are inactive (reviewed by [46,47]). This shuttling of the CHT-1 is a relatively slow process, and, as a consequence, HACU activity in the synaptic membrane can be measured ex vivo [43]. Even when synaptosomes are isolated in a procedure that takes several minutes, the synaptic HACU values reflect cholinergic firing rate in vivo. For instance, the rate of HACU in the hippocampus will be reduced when rats were pretreated with barbiturates (which reduce cholinergic firing) but it will increase when the cholinergic terminals fired rapidly, e.g., after seizures [43]. In short, HACU activity follows the firing rate of cholinergic neurons and reflects central cholinergic activity. This characteristic has often been interpreted to show that HACU is the major rate-limiting step for ACh synthesis, provided that the other members of the cholinergic synapse (ChAT, VAChT and AChE) are intact and work at normal activity levels.

As with ChAT, deletion of the CHT-1 gene in mice is lethal immediately after birth because of respiratory failure. Mice hemizygous for CHT-1 are fertile and appear normal [41]. Closer examination of these mice revealed higher tissue levels of choline in the brain, but lower levels of ACh corroborating the role of the transporter as choline carrier [48]. Mice showed normal behavior but weaker performances when challenged behaviorally or pharmacologically [39]. Peripherally, mice deficient in CHT-1 have tachycardia and ventricular dysfunction, and they have lower motoric capacity while mice overexpressing CHT-1 have increased motor endurance and less fatigue [49]. Mutations in men have similar consequences. Asians and Ashkenazi Jews have relatively frequent mutations that reduce CHT-1 activity, but serious health consequences have not been reported [40,50]. Inactivating mutations are also lethal in humans [51].

### 2.6. The Vesicular Acetylcholine Transporter VAChT

The transporter for ACh into cholinergic vesicles, VAChT (SLC18A3), is a large glycoprotein that exchanges ACh for protons. The binding and transport mechanisms have been elucidated by cryo-electron microscopy [52]. VAChT is of pivotal relevance for the functioning of the cholinergic terminals. Inactivating mutations of VAChT are lethal after birth. Mice with partial deficits of the VAChT gene are viable; at 65% reduction in VAChT, neuromuscular problems are evident but at 45% reduction, mice moved normally and could be investigated in behavioral paradigms [53]. These mice showed a significant reduction in cortical ACh release under basal and stimulated conditions and an impairment of cognitive functions (e.g., social recognition; reviewed in [54]). The mechanism of ACh release seemed unchanged, and the cholinergic deficit could be corrected by AChE inhibition.

VAChT has a low Km for ACh (approx. 1 mM) and is known to be a slow transporter, and the present data demonstrate that lower levels of VAChT limit cholinergic function due to reduced storage and release of transmitter. In agreement with mouse data, mutations of VAChT in humans lead to myasthenic syndromes [55]. Vice versa, in mice that contained multiple copies of the VAChT gene and displayed increased VAChT expression, ACh release from hippocampal slices was increased [56]. VAChT, therefore, controls the filling of vesicles with ACh but does not interfere with release processes.

### 2.7. Acetylcholinesterase (AChE)

AChE, the enzyme that terminates the action of ACh, is one of the fastest known enzymes; one molecule of protein hydrolyzes 5–6000 molecules of ACh per second. It is widely distributed in the brain [8]. During development, AChE is expressed before the other cholinergic genes occur, and a non-cholinergic role, e.g., in neuronal migration has been suggested [57]. AChE is not appreciably regulated by phosphorylation, and there are few mutations in the human gene, usually with very mild consequences on enzymatic activity. Large-scale inhibition of AChE is very toxic to any organism (see below), and knockout of the AChE gene is lethal in fruit flies and zebrafish, but not in mice (see below). It should be noted that AChE activity is not easy to measure because many external factors, e.g., the presence of various detergents, strongly influence enzymatic activity [58].

AChE occurs in multiple subtypes, for instance coupled to GPI anchors in erythrocytes or as a 12mer coupled to collagen Q in the neuromuscular junction. In the CNS where AChE is primarily formed by cholinergic neurons, the majority of AChE molecules occur as tetramers bound to the synaptic membrane by interaction with PRiMA, the proline-rich membrane anchor [59]. AChE can also exist as a monomeric form which can be found in soma and dendrites [60]. A well-studied response is the increase in AChE expression upon exposure to AChE inhibitors. Increased ACh (via muscarinic receptors) increases AChE expression and formation of a stress-induced, “read-through” form of AChE that occurs as a monomer [61]. This response can also be provoked by psychological stress or by head injury and lasts for several weeks reducing cholinergic signaling in the CNS [61]. The control of AChE expression, e.g., by miR-132, was widely investigated [62,63]. Like ACh, AChE also occurs in non-neuronal cells; its potential roles in neural development, synaptogenesis and immune function require further research [57,63].

Inhibition of AChE is a very common phenomenon. Numerous AChE inhibitors have been described in the plant kingdom, and inhibition of AChE is arguably the most common defense of plants against mammals [3]. Common insecticides as well as nerve gases have anti-AChE activities, but some AChE inhibitors also have therapeutic use, including the common anti-dementia drugs donepezil (synthetic), rivastigmine (developed from physostigmine, a plant product) and galanthamine (a plant product) [64].

While AChE inhibitors are lethal in high does, mice that are deficient in AChE surprisingly survive although they are small and seizure-prone and have weak muscles [65]. However, with adequate (liquid) food, they can survive for several months. ACh levels are increased manifold [38], but the mice survive because of the presence of butyrylcholinesterase (BChE).

It should be mentioned that synapses do not only consist of pre- and postsynaptic neurons but are also enclosed by glial cells that contribute to the dynamics of local metabolism. BChE, for example, is formed by Schwann cells in the neuromuscular junctions where its contributions to ACh hydrolysis were intensively investigated [66]. In the CNS, BChE is formed by astrocytes, and it is able to partly substitute for AChE in ACh hydrolysis [67]. However, in the CNS, BChE does not make a contribution when AChE is active [68]. Further roles of astrocytes in ACh metabolism are poorly documented, except that they are likely to participate in uptake and release of choline [18].

BChE likely evolved to protect mammals against plant-derived AChE inhibitors [3]. It is present in high amounts in blood and is often called plasma cholinesterase. Humans harbor many mutations in BChE and, consequently, have variable responses towards AChE inhibitors, this includes some clinically used inhibitors [69].

Mice that lack one functional allele of AChE appear normal although they are more sensitive to AChE inhibitors. They have only approx. 50% of brain AChE activity and approx. double the amount of extracellular ACh. Further analysis of several mouse species revealed that the extracellular ACh level in mice is inverse to AChE activity, i.e., ACh level = 1/AChE activity [68]. This means that a 50% inhibition of AChE activity would be required to double the endogenous ACh level in the brain. This value is seldom reached by drug treatment [70].

### 2.8. Plasticity of Cholinergic Presynaptic Mechanisms

Under normal circumstances, central cholinergic terminals release ACh based on axonal impulse flow which is controlled by neuronal network activity. Glucose and choline are usually in good supply but can become rate-limiting under specific conditions, e.g., during mitochondrial dysfunction in ischemia, but also when neurons are firing rapidly [18,23]. ChAT activities are not usually rate-limiting, nor is VAChT transport activity, although VAChT deficits reduce loading of ACh into vesicles [53,54]. As discussed above, HACU (mediated by CHT-1) effectively adapts to the cholinergic firing rate by increasing or decreasing its membrane location on a short time scale (minutes) [43,46]. Studies in several transgenic mouse strains revealed cholinergic plasticity that relied on changes in HACU activity. For instance, hemizygous animals that are 50% deficient in ChAT appear largely normal, and ACh release is unchanged [15]. However, they have increased HACU activity, which suggests that they compensate for low ChAT activity with faster firing and higher ACh turnover [15]. On the other hand, mice with an overexpression of AChE suffer from a reduction in ACh levels that is partially compensated by an increase in HACU and ACh synthesis [71]. Taken together, these examples illustrate an impressive adaptability of central cholinergic fibers to stressful conditions (resilience) [72].

A remarkable example of cholinergic plasticity was reported in a transgenic mouse strain that lacked PRiMA, the membrane anchor of AChE in the brain [59]. PRiMA deficiency reduces AChE activity by 90–95% and increases ACh levels 5–10-fold, yet the mice appear normal [73] and do not show signs of cholinergic over-stimulation, such as poor muscle function or seizures. Among the compensatory changes are dysfunctional M2-receptors [74]. Down-regulation of receptors may be an additional pathway how mice adapt to unphysiologically high ACh levels.

### 2.9. Muscarinic ACh Receptors

After release from cholinergic terminals, the actions of ACh are mediated via metabotropic (G-protein coupled) muscarinic and ionotropic nicotinic receptors (see Table 1 for main subtypes in the brain). All five subtypes of muscarinic receptors (mAChR) occur in the brain. Muscarinic M1, M3 and M5 receptors are largely excitatory, lead to the formation of inositol triphosphate, increase calcium in postsynaptic cells and cause a long-lasting reduction of potassium currents enhancing neuronal excitability (M current) [75]. The M1 receptor is the main excitatory receptor in the brain, it is present in post-synaptic membranes on, for instance, GABAergic interneurons and glutamatergic pyramidal neurons. M3 receptors in the hypothalamus are involved in appetite control. M5 receptors contribute to reward pathways and cerebral blood flow [75].

M2 and M4 receptors are inhibitory receptors; they reduce cAMP and calcium influx and increase potassium conductance, thereby hyperpolarizing neurons. M4 receptors predominate in the striatum where they reduce the activity of dopaminergic neurons. Hippocampal and cortical areas mainly express M2-type muscarinic receptors that, among other things, inhibit GABAergic transmission [75]. Both M2- and M4-type receptors also act as presynaptic inhibitory feedback receptors for ACh release [76]. It must be noted that inhibitory feedback via M2/M4 receptors is weak under basal conditions; they limit ACh release only when ACh levels are increased, e.g., in the presence of AChE inhibitors. From microdialysis studies, it is known that, at higher levels of AChE inhibition, muscarinic antagonists (usually atropine or scopolamine) cause a several-fold increase in ACh release, which is not seen under basal conditions [77,78].

### 2.10. Nicotinic ACh Receptors

Nicotinic receptors (nAChR) are much less abundant in the brain than muscarinic receptors but have high functional significance. Among the several subtypes present in the brain, the ɑ4ß2 subtype is the most important, because it mediates most of actions of ACh in the brain, e.g., addiction to nicotine in the reward system or appetite suppression in the hypothalamus [1,2,3] (see also Table 1). ɑ4ß2 nicotinic receptors have high affinity for ACh and cause strong stimulatory effects on the release of catecholamines (dopamine and noradrenaline) and of GABA while ACh release is less prominently affected [79]. Other nicotinic subtypes, e.g., ɑ6- and ß3-subunits, are expressed in several brain regions including the striatum and increase the variability of nAChR in the brain [80]. In addition, the homomeric ɑ7-nicotinic receptor is of interest because it has an increased calcium permeability and activates, for instance, glutamatergic responses in hippocampus and cortex [81]. Choline in high concentrations is an agonist at nicotinic ɑ7-receptors; however, homeostatic mechanisms such as cellular choline uptake and rapid release from the CSF back into the blood make it unlikely that choline reaches the necessary concentrations (>100 µM) that are required for ɑ7-receptor activation [18]. Nicotinic receptors containing ɑ3- und ß4-subunits are prominent in the autonomic nervous system but have a lower expression in the brain [80]. Overall, nicotinic receptors are excitatory by nature (mediating sodium influx) but the functional response of neurons to nicotinic activation depends strongly on the state of neurons under study [1]. In addition, rapid desensitizations of nicotinic responses must be considered [79,80].

### 2.11. Control of ACh Release by Presynaptic Receptors

In addition to cholinergic receptors (see above), ACh release is also influenced by many other neurotransmitters, as shown in numerous studies in brain slices, synaptosomes and in vivo by microdialysis [82,83]. As a rule, centrally depressing drugs such as benzodiazepines and anesthetic gases, but also adenosine, reduce ACh release at cholinergic synapses. Increases in ACh release are often seen with glutamate, ATP and amines such as serotonin [84]. Striatal cholinergic interneurons show a different behavior, they are activated via glutamatergic fibers and NMDA receptors which are coupled to NO synthesis [85], but they are inhibited by dopaminergic fibers, which activate presynaptic D2 receptors on striatal cholinergic terminals (D1 receptors are facilitatory) [86].

### 2.12. Cholinergic Systems and Neurological Disease

Among diseases of the brain, deficits in central cholinergic signaling were observed most prominently in Alzheimer’s disease (AD). However, cholinergic deficits are also observed in dementia with Lewy bodies and Down syndrome but not in frontotemporal dementia [2,87]. Cholinergic cell death also occurs in Parkinsons’s and Huntington’s disease, and epileptic seizures up-regulate ACh release during status epilepticus [88]. Affective disorders also have a cholinergic component. Both muscarinic and nicotinic agonists were shown to alleviate symptoms of schizophrenia, while the blockade of muscarinic receptors, e.g., by scopolamine is beneficial in depression [83]. The reason for the selective vulnerability of central cholinergic neurons remains elusive [89], and novel treatment approaches are still in the testing stage [90].

Alzheimer’s disease is the most intensively investigated neurodegenerative disease, with hallmarks such as amyloid deposition, tau aggregation, energy failure and neuroinflammation serving as potential therapeutic targets [64,70]. However, treatments of neuropathological hallmarks such as amyloid deposition had limited success up to now [91]. Tau aggregation remains a viable target as tau-transgenic mice have cholinergic dysfunction [92], and treatment of energy failure and mitochondrial dysfunctions may be influenced by adequate nutrient and vitamin intake [93].

Cholinergic fibers degenerate early in AD, and this phenomenon has gained wide popularity because deterioration of cholinergic function correlates with clinical signs of confusion and memory loss [64,70]. Much effort has been invested in finding a connection between amyloid formation and cholinergic cell death, and practically all cholinergic functions discussed in this article were postulated to interact with (intra- or extracellular) amyloid peptides [94]. In amyloid-bearing mice, reductions in ACh release were found in several strains (e.g., [95]), but no major cholinergic dysfunction was seen in mice with lower amyloid burden [78,96]. More recently, transgenic rat models of AD that more closely mimic the human situation were described [97,98].

Irrespective of the elusive etiology of Alzheimer’s disease, central cholinergic dysfunction remains the most druggable characteristic of the disease [64]. AChE inhibitors delay the breakdown of Ach, thereby increasing cholinergic tone, while receptor agonists directly induce muscarinic or nicotinic signaling. Nicotinic agonists, however, suffer from possible addictive properties and have not been successfully developed. Muscarinic agonists have been developed, but the muscarinic agonist xanomeline was first introduced as a drug to treat schizophrenia [76]. Of note, full agonists stimulate receptors for many hours and do not reflect the in vivo-situation in which ACh is released on a sub-second time scale and the activation of cholinergic receptors is dynamic [99]. A possible solution to this problem may be the development of partial muscarinic agonists or muscarinic PAMs, i.e., partial allosteric agonists that only facilitate muscarinic receptor activation when ACh is present [70,76].

At present, inhibitors of AChE are still the mainstay of AD therapy. However, pro-cholinergic therapy has adverse side effects in peripheral systems (mainly the vagal nerve) causing, for instance, nausea, diarrhea and bradycardia. At tolerable doses, PET studies showed that 20–40% of brain AChE activity are inhibited in patients [70]. Judging from mouse studies in which ACh levels are inversely correlated with AChE activity (see above; [68]), this inhibition would cause less than a two-fold increase in brain ACh levels, which may be insufficient to improve AD symptoms. Recent attempts to improve AChE inhibitor therapy include high-dose therapy with slow-release formulations or the combination of centrally active AChE inhibitors with non-brain permeable, only peripherally acting muscarinic antagonists to reduce systemic toxicity of AChE inhibitors [100].

Finally, cholinergic synapses are also the target structures of botulinum toxin (BTX), a mixture of clostridial toxins that are considered the most toxic molecules on the planet. BTX binds to cholinergic motor neurons with high affinity, due to the presence of certain gangliosides and synaptic vesicle protein 2 (SV-2) [101,102]. After entering the synaptic fluid, the toxins act as metalloproteases to cleave proteins required for transmitter release, such as SNAP-25, VAMP and syntaxin. The consequence is flaccid paralysis and death due to loss of respiration. In small doses, BTX can be used to treat dystonia, spasticity and (possibly) neuropathic pain. In addition to motor neurons, BTX also attack cholinergic fibers in the autonomic nervous system, but they do not cross the blood–brain barrier. Central functions of BTX, even after peripheral uptake, have been reported, but the involvement of central cholinergic synapses in BTX action remains to be investigated.

## 3. Conclusions

Central cholinergic neurons express cholinergic genes that determine cholinergic signaling. Severe impairments of cholinergic functions are not compatible with life; loss of ChAT, VAChT or CHT-1 activity in mice causes early death after birth. In humans, mutations of cholinergic genes have been described that lower cholinergic activity; consequences include myasthenic syndromes, difficulties of breathing and possibly some central effects, such as attention disorders or hyperactivity. Cholinergic deficits are particularly prominent in Alzheimer’s disease and inhibition of AChE is the mainstay of therapy.

While much knowledge on central cholinergic mechanisms has been obtained, several features remain insufficiently explored. Numerous microdialysis studies have revealed regulations of ACh release under various conditions, but methodical limitations limit our understanding of rapid responses. Progress in the development of biosensors [39,72] and, more recently, in genetically encoded fluorescent sensors coupled with fiber photometry promise a better understanding of rapid cholinergic responses [103]. Studies on the ontogeny of the cholinergic system are proceeding [7] but further work is required to understand the regulation of the cholinergic gene locus. Non-invasive techniques will allow screening of human central cholinergic systems, although current techniques (e.g., the “choline” peak in fMRI) are not yet reliable and may even be misleading [27]. Future studies should focus on a deeper understanding of cholinergic plasticity during development, aging and dementia and uncover novel ways of cholinergic manipulations, e.g., by RNA-based therapies [104]. Given the involvement of central cholinergic systems in numerous brain diseases, the future of cholinergic research appears promising.

## Figures and Tables

**Table 1:** Main cholinergic receptors in the CNS.

Muscarinic Receptors	2nd Messenger	Location and Function
M1, (M3, M5)	↑ IP_3_, ↑ Ca^2+^, ↓ K^+^	Excitatory receptors, M1 ubiquitous in forebrain
M2, M4	↓ cAMP, ↓ Ca^2+^; ↑ K^+^	Main presynaptic receptors, reduce transmitter release
**Nicotinic receptors**		
ɑ4ß2-Receptors	↑ Na^+^	Main excitatory receptor, releases catecholamines
ɑ7-receptors	↑ Na^+^, ↑ Ca^2+^	Releases glutamate in hippocampus

## Abbreviations

The following abbreviations are used in this manuscript:

ACh	Acetylcholine
AChE	Acetylcholinesterase
BChE	Butyrylcholinesterase
ChAT	Choline acetyltransferase
HACU	High-affinity choline uptake
mAChR	Muscarinic ACh receptor
nAChR	Nicotinic ACh receptor
PC	Phosphatidylcholine
VAChT	Vesicular acetylcholine transporter
